# Comparative Transcriptome Analysis of Purple and Green Flowering Chinese Cabbage and Functional Analyses of *BrMYB114* Gene

**DOI:** 10.3390/ijms241813951

**Published:** 2023-09-11

**Authors:** Mei Fu, Juxian Guo, Kang Tang, Shizheng Jiang, Shanwei Luo, Wenlong Luo, Imran Khan, Guihua Li

**Affiliations:** 1 Guangdong Key Laboratory for New Technology Research of Vegetables, Vegetable Research Institute, Guangdong Academy of Agricultural Sciences, Guangzhou 510642, China; fumei@gdaas.cn (M.F.); guojuxian@gdaas.cn (J.G.); 20203137146@stu.scau.edu.cn (K.T.); jiangshizheng2021@163.com (S.J.); xuan7355@163.com (S.L.); luowenlong@gdaas.cn (W.L.); 2 College of Horticulture, South China Agricultural University, Guangzhou 510642, China

**Keywords:** transcriptome, flowering Chinese cabbage, anthocyanin, *BrMYB114*

## Abstract

Flowering Chinese cabbage (*Brassica rapa* var. *parachinensis*) is one of the most popular vegetables in the south of China. As an antioxidant, anthocyanin is an important quality trait in vegetables, and the gene related to anthocyanin biosynthesis in purple flowering Chinese cabbage is also important. In this study, two flowering Chinese cabbage with extreme colors in the stem were used as materials for transcriptome analysis. RNA-seq analysis showed that 6811 differentially expressed genes (DEGs) were identified, including 295 transcription factors. Phenylpropanoid biosynthesis, flavone and flavanol biosynthesis, and flavonoid biosynthesis pathways were found to be significantly enriched in the purple flowering Chinese cabbage. A total of 25 DEGs associated with anthocyanin biosynthesis were found at a higher expression in purple flowering Chinese cabbage than in green flowering Chinese cabbage. Bioinformatics analysis shows that *BrMYB114* is a candidate gene for the regulation of anthocyanin biosynthesis, and heterologous expression analysis of *BrMYB114* in *Nicotiana benthamiana* indicates that *BrMYB114* functions in anthocyanin biosynthesis. Therefore, our findings provide vital evidence for elucidating the molecular mechanism in the purple stem in flowering Chinese cabbage.

## 1. Introduction

Nutritious, low-cost vegetables are needed to meet consumer demands and improve human health. Flowering Chinese cabbage (*Brassica rapa* var. *parachinensis*), a *Brassica* plant, has a long history of cultivation and includes numerous germplasms with complex backgrounds. This plant has recently become a global crop due to its gradual introduction into Southeast Asia, Europe, and the Americas [1]. Many *Brassica* plants have green stems, but there are also some varieties with purple stems and white stems [1,2,3]. The purple phenotype in *Brassica* plants is due to the differential accumulation of pigments, such as chlorophyll, carotene, and anthocyanins. Among these, anthocyanins have received the greatest attention as secondary metabolites that can influence leaf color [4]. Plants with blue, purple, violet, and red pigmentation contain a high number of anthocyanins [5]. Anthocyanins are water-soluble flavonoid pigments with considerable antioxidant effects [6]. These compounds function in plant reproduction by attracting pollinators and seed distributors and protecting against various environmental and biotic stresses. Therefore, developing purple-stem *Brassica* varieties has become one of the most important breeding objectives of *Brassica* breeders.

More than 600 different types of anthocyanins have been identified so far, of which six are abundant in plants. The accumulation patterns of anthocyanins affect changes in the coloration of plants. The biosynthetic pathway responsible for anthocyanin production has been elucidated in previous studies. In the first step, phenylalanine is converted into cinnamic acid by phenylalanine ammonia-lyase (PAL). Then, cinnamic acid was converted into different types of anthocyanin by several enzyme activities, which include 4-coumarate-CoA ligase (4CL), chalcone synthase (CHS), chalcone isomerase (CHI), dihydroflavonol 4-reductase (DFR), and anthocyanin synthase (ANS). The ability of structural genes to increase anthocyanin biosynthesis in plants is largely dependent on transcription factors [7].

Anthocyanin biosynthesis is transcriptionally regulated by a complex of MYB-bHLH-WD40 transcription factors [8]. Among these, various R2R3-MYB transcription factors activate anthocyanin biosynthesis [9]. For example, PpMYB10 and PpMYB114 are important regulators of anthocyanin biosynthesis in pear (*Pyrus bretschneideri*) [10]. In Arabidopsis (*Arabidopsis thaliana*), AtMYB75 (PAP1) and AtMYB90(PAP2) activate the expression of downstream structural genes involved in anthocyanin biosynthesis [11,12,13]. SIMYB75 affects the quality of tomatoes (*Solanum lycopersicum*) by regulating anthocyanin biosynthesis [14]. bHLH transcription factors interact with MYB and WD40 transcription factors to regulate the expression of *CHS*, *DFR*, and *UFGT* in asparagus (*Asparagus officinalis*, L.) [15]. WD40 transcription factors do not bind directly to the promoter of structural genes; instead, regulating their expression by forming a complex with MYB and bHLH transcription factors [16,17]. Other transcription factors also affect anthocyanin biosynthesis, such as WRKY [18], ERF [19], NAC [20], bZIP [21], and BBX [22].

In this study, the transcriptomes of purple and green flowering Chinese cabbage were used to identify differentially expressed genes (DEGs) in anthocyanin biosynthesis. Anthocyanin synthesis-related transcripts were found to be significantly upregulated in purple Pak-Choi (*Brassica campestris* L. ssp. *chinensis* L. *Makino*) [23]. Furthermore, two non-heading Chinese cabbages (*Brassica campestris* ssp. *Chinensis*) were used for RNA-seq, and the results suggested that BcTT8 plays an important role in the process of anthocyanin synthesis in non-heading Chinese cabbages [24]. Previous studies have been conducted on the molecular regulation of anthocyanins in *Brassica* [25,26,27], and some research progress has been made on the color of flowering Chinese cabbage. For example, QTL-Seq and sequence assembly mapped three genes for the purple trait in Zicaitai [1,3,28], and transcriptome revealed BrTCP15 participates in the biosynthesis of anthocyanins under low-light conditions [29]. The molecular mechanism of anthocyanin biosynthesis has not yet been fully elucidated. Therefore, the aim of the present study is to investigate the molecular mechanisms of anthocyanin biosynthesis in two flowering Chinese cabbage varieties, a green inbred line Futiancaixin, and a purple inbred line Zicaitai.

## 2. Results

### 2.1. RNA Sequencing Analysis

**Figure 1:**
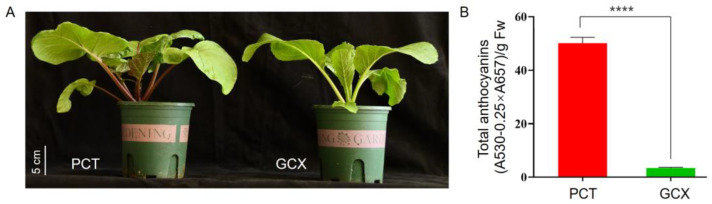
The phenotypes, and anthocyanin content of purple flowering Chinese cabbage (Zicaitai, PCT), and flowering Chinese cabbage (Futiancaixin, GCX): (**A**) phenotypes of PCT and GCX; (**B**) anthocyanin content of PCT and GCX. Each column represents the mean ± SD (*n* = 3). Asterisks indicate a significant difference (Student’s *t*-test, ****, *p* < 0.0001).

To explore the molecular mechanism of anthocyanin biosynthesis, transcriptome sequencing of PCT and GCX was performed (Figure 1A), and the content of anthocyanidin in these two varieties was greatly different (Figure 1B). Six libraries were constructed from these two varieties, and a total of 39.13 GB of clean data were obtained after filtering low-quality reads. The Q30 values of all samples were ≥92.11%. The GC content ranged from 40% to 50% (Table S1). The clean data were mapped to the reference genome with a mapping ratio of >80.93% (Table S2). Principal component analysis (Figure S1A) and correlation coefficient analysis (Figure S1B) confirmed the similarities of the expression patterns among different replicates under the same treatment. These data indicate that the sequencing quality was reliable. We identified a total of 6811 differentially expressed genes (DEGs) in PCT and GCX, which included 3547 upregulated and 3264 downregulated genes (Table S3).

### 2.2. Functional Annotation and Classification of the DEGs

**Figure 2:**
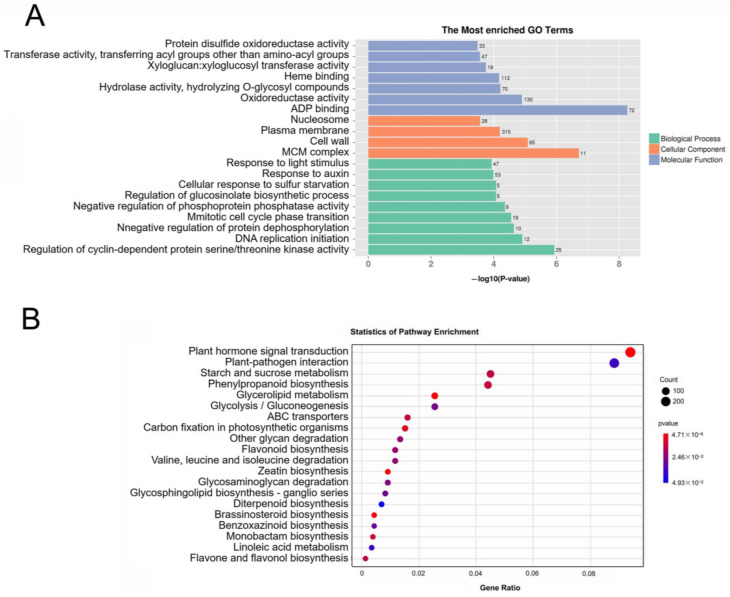
GO enrichment and KEGG enrichment of DEGs among PCT and GCX: (**A**) GO enhancement of DEGs among PCT and GCX; (**B**) KEGG enhancement of DEGs among PCT and GCX.

Homology analyses were performed against public databases, including GO, KEGG, COG, KEGG Ortholog (KOG), NR, NOG, PFAM, and SWISS-PROT, to determine the putative functions of the assembled genes. This identified a total of 265 significantly enriched GO terms (Table S4), including 145 GO terms in the biological process’s category, such as responses to the light stimulus (GO:0009416), response to auxin (GO:0009733), and regulation of glucosinolate biosynthetic process (GO:0010439). By contrast, 98 GO terms were in the molecular function category, including ADP binding (GO:0043531), hydrolase activity, hydrolyzing O-glycosyl compounds (GO:0004553), oxidoreductase activity (GO:0016491), and heme binding (GO:0020037). Twenty-two significantly enriched GO terms were in the cellular components category, including the terms MCM complex (GO:0042555) and cell wall (GO:0005618) (Figure 2A). We then mapped the DEGs to KEGG pathways to further explore their biological roles; specifically, 2293 DEGs were mapped to 135 KEGG pathways (Table S5). Among these pathways, plant hormone signal transduction (Ko04075), flavonoid biosynthesis (ko00941), flavone and flavanol biosynthesis (ko00944), glycolipid metabolism (Ko00561), and brassinosteroid biosynthesis (Ko00905) were significantly enriched (Figure 2B). Therefore, pigment-related pathways were significantly enriched among the DEGs.

### 2.3. Key Genes Related to the Anthocyanin Biosynthetic Pathway

**Figure 3:**
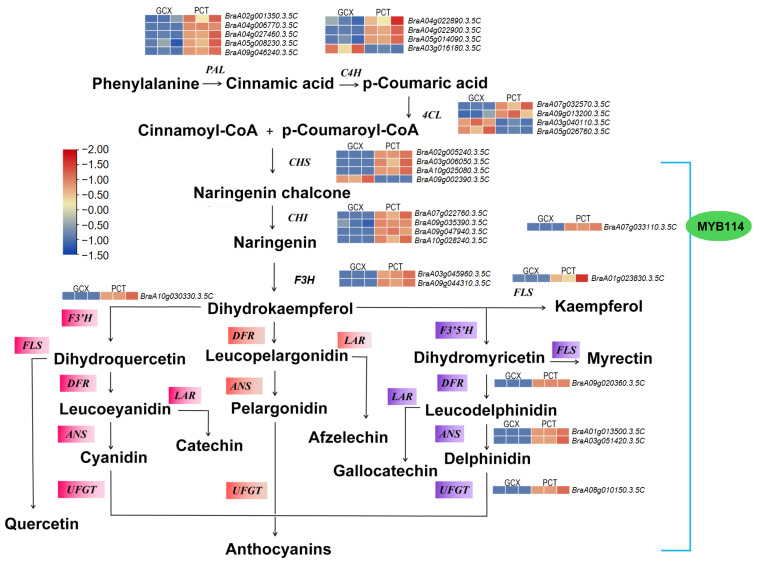
Schematic presentation of biological pathways involved in the accumulation of anthocyanin biosynthesis in PCT. Six boxes next to the structural gene indicate the heat map expression level of structure gene in PCT and GCX, the color scale from blue to red represent the expression of structural gene from low to high. Three different colored boxes (pink, orange, and purple) in the bottom half of the picture represent the biosynthetic pathways of three different anthocyanins.

To explore the differences in anthocyanin biosynthesis between the two varieties, we identified DEGs related to the anthocyanin biosynthesis pathway; specifically, the phenylpropanoid biosynthesis (Ko00940), flavone, and flavanol biosynthesis (Ko00944), flavonoid biosynthesis (Ko00941), and anthocyanidin biosynthesis (Ko00942). Twenty-nine structural genes were differentially expressed between varieties (Figure 3), including 5 *PAL*(*BraA02g001350.3.5C*, *BraA04g006770.3.5C*, *BraA04g027460.3.5C*, *BraA05g008230.3.5C*, *BraA09g046240.3.5C*); 4 *C4H*(*BraA04g022890.3.5C*, *BraA04g022900.3.5C*, *BraA05g014090.3.5C*, *BraA03g016180.3.5C*); 4 *4CL*(*BraA07g032570.3.5C*, *BraA09g013200.3.5C*, *BraA03g040110.3.5C*, *BraA05g026760.3.5C*). The early biosynthesis contained 4 *CHS*(BraA02g005240.3.5C, *BraA03g006050.3.5C*, *BraA10g025080.3.5C*, *BraA09g002390.3.5C*); 4 *CHI*(*BraA07g022760.3.5C*, *BraA09g035390.3.5C*, *BraA09g047940.3.5C*, *BraA10g028240.3.5C*); 2 *F3H*(*BraA03g045960.3.5C*, *BraA09g044310.3.5C*); 1 *FLS*(*BraA01g023830.3.5C*). The later biosynthesis contained 1 *F3′H*(*BraA10g030330.3.5C*); 1 *DFR*(*BraA09g020360.3.5C*); 2 *ANS*(*BraA01g013500.3.5C*, *BraA03g051420.3.5C*) genes and 1*UFGT*(*BraA08g010150.3.5C*). Twenty-five structural genes were expressed at higher levels in PCT than in GCX, which is consistent with the purple coloration of PCT.

### 2.4. Verification of Gene Expression Profiles by qRT-PCR

**Figure 4:**
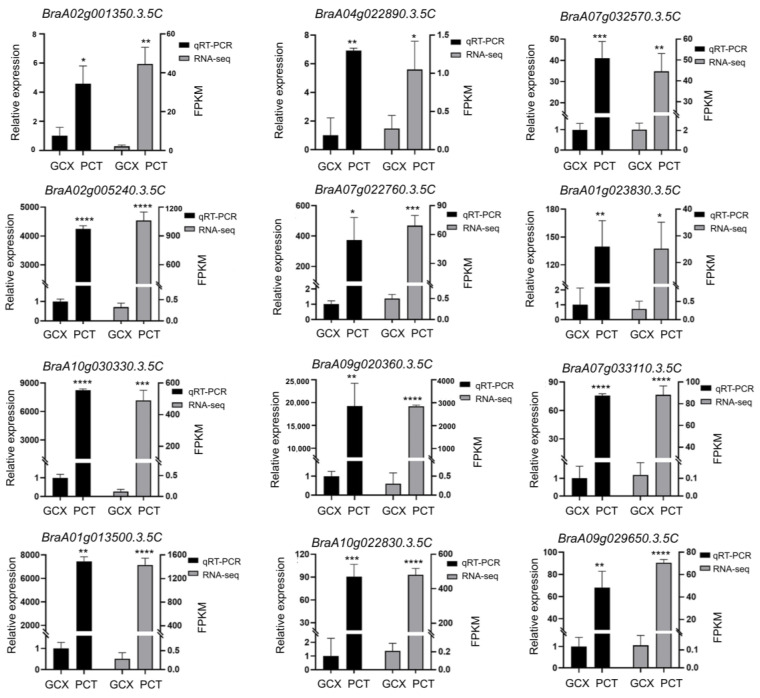
An evaluation of the RNA-seq results with qRT-PCR. The values represent the means ± SD of FPKM data and qRT-PCR data of 3 biological replicates, *, **, ***, and **** indicate significant difference at *p* < 0.05, *p* < 0.01, *p* < 0.001, and *p* < 0.0001 based on Student’s *t*-test, respectively. FPKM is the expected number of fragments per kilobase of transcript sequence per million base pairs.

To verify the accuracy and reliability of the transcriptome data, we selected 12 DEGs associated with flavonoid, phenylpropanoid, and anthocyanin biosynthesis and measured their expression levels in both GCX and PCT through performing qRT-PCR. The expression patterns of the unigenes analyzed through qRT-PCR (*BraA02g001350.3.5C*, *BraA04g022890.3.5C*, *BraAo7g032570.3.5C*, *BraA01g013500.3.5C*, *BraA10g022830.3.5C*, *BraA02g005240.3.5C*, *BraAo7g022760.3.5C*, *BraA01g023830.3.5C*, *BraA10g030330.3.5C*, *BraA09g020360.3.5C*, *BraA07g033110.3.5C*, *BraA09g029650.3.5C*) were constant with the FPKM values attained by RNA-seq (Figure 4), confirming the reliability of our data.

### 2.5. Transcription Factor Analysis of Flowering Chinese Cabbage

**Figure 5:**
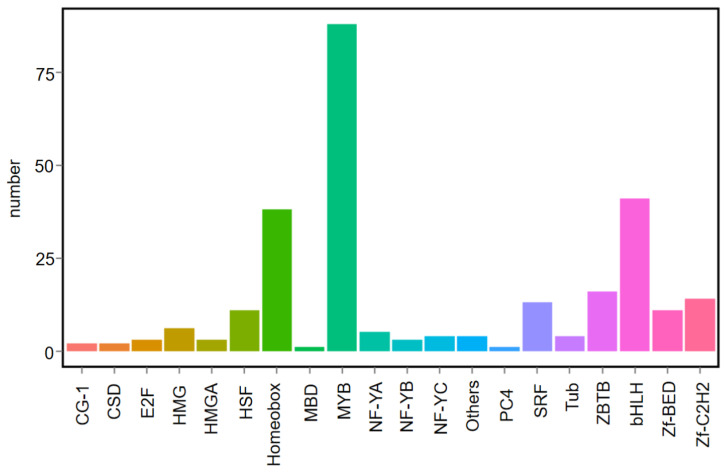
Differentially expressed transcription factor encoding genes between PCT and GCX.

TFs are responsible for regulating anthocyanin biosynthesis in plants. The transcripts of transcription factors encoding genes were further analyzed using PCT in order to uncover the regulatory mechanisms driving this significant upregulation of anthocyanin and flavonoid biosynthesis genes. In total, 295 transcription factors encoding genes were differentially expressed (Table S6). MYB and bHLH were identified as the most abundant transcription factors, and other TFs were also identified (Figure 5). MYB transcription factors are important regulators of anthocyanin biosynthesis. Transcriptome analysis showed that *BrMYB114* (*BraA07g033110.3.5C*) is expressed at higher levels in PCT than in GCX (Figure 3). According to the *BrMYB114* homologous clone results, nucleotide sequence alignment shows that *BrMYB114* is highly similar to *BrMYB2* (*Bra004162*) (Figure S2), which is a transcription factor involved in anthocyanin biosynthesis in purple-headed Chinese cabbage [26,27]. Sequence analysis showed that *BrMYB114* and *BrMYB2* share the same coding sequence (Figure S2). There is a possibility that *BrMYB114* plays a role in flowering Chinese cabbage plants having different-colored leaves.

### 2.6. Heterologous Expression Analysis of BrMYB114 in N. benthamiana

**Figure 6:**
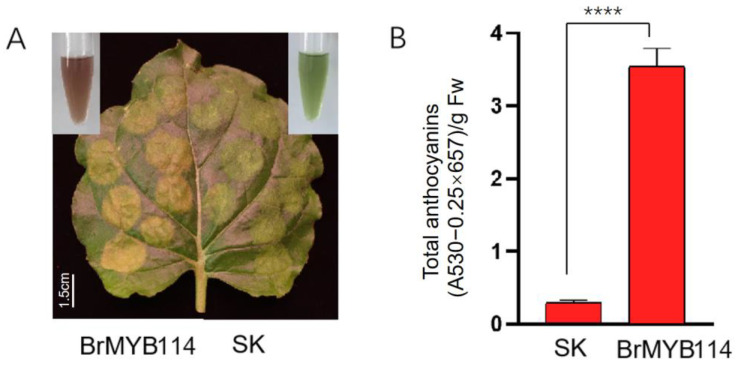
Overexpression of *BrMYB114* using *N. benthamiana* transient expression system: (**A**). color changes induced by transiently expressing *BrMYB114* in *N. benthamiana*. Anthocyanin was extracted 7 days after infiltration; (**B**). total anthocyanin content measured in the infiltration patches in (**A**). Data are means ± SD obtained from three biological repeats. Asterisks indicate a significant difference (Student’s *t*-test, ****, *p* < 0.0001.

BrMYB2 promotes anthocyanin production in line 11S91 [26,27], and *BrMYB114* was expressed at higher levels in PCT than in GCX, as indicated by transcriptome analysis. To explore whether *BrMYB114* participates in anthocyanin accumulation in PCT, we overexpressed *BrMYB114* in *N. benthamiana*. Seven days later, anthocyanin was extracted from leaves of *N. benthamiana* overexpressing *BrMYB114*, the colour of extract showed red. The results of anthocyanin detection showed that leaves of *N. benthamiana* overexpressing *BrMYB114* had a higher anthocyanin content than control leaves transformed with the empty expression vector (Figure 6A,B). These results indicate that BrMYB114 functions in anthocyanin biosynthesis in PCT.

## 3. Discussion

Flowering Chinese cabbage has high nutritional value, making it an important vegetable crop. The higher nutritional component content in purple-leaved vegetables, such as anthocyanins, has attracted more attention. In order to produce food with health benefits, the extraction of anthocyanin from colored vegetables can be altered into functional components. In *Brassica* vegetables, transcriptome analysis is an effective method for selecting DEGs, which helps identify candidate genes [23,25,30,31,32]. Research is needed to understand why flowering Chinese cabbage have purple stems. Using transcriptome analysis, the present study sought to understand how flowering Chinese cabbage stems develop their color.

According to the transcriptome analysis of two non-heading Chinese cabbages, DEGs were found in purple non-heading Chinese cabbage to be enriched in the anthocyanin biosynthesis pathway, flavonoid pathway, sucrose and starch metabolism pathway, and the secondary metabolite pathway [24]. Similarly, we found the same enrichment pathway in this study (Figure 2). Several genes involved in the phenylpropane and flavonoid pathways are involved in the synthesis of anthocyanin, a subgroup of flavonoid [33], so genes related to phenylalanine metabolism and flavonoid metabolism have been significantly enriched in PCT. Several enzymes are required to catalyze anthocyanin synthesis, and starch hydrolysis costs a considerable amount of energy to encode these products [34]. Therefore, it is understandable that the genes associated with the sucrose and starch metabolism pathway have been highly significantly accumulated in PCT. Our analysis follows the transcriptome profiling that was conducted on non-heading Chinese cabbage [24].

There are numerous outlines of evidence suggesting that *PAL, CHI*, *C4H*, and *4CL* play an important role in anthocyanin synthesis in the initial stages, and *F3′5′H*, *LODX*/*ANS*, *F3′H*, *UFGT*/*3GT*, and *DFR* play an important part in upcoming stages. To synthesize all three branches of anthocyanins and proanthocyanidins, a functional gene called *F3H* accelerates the formation of dihydroflavonol from substrates (Figure 3). It was found that high *F3′H* expression promoted both anthocyanin and flavonoid accumulation [35]. By binding both *DFR* and *UFGT*, *F3′H* plays a vital role in enhancing the production of dihydroquercetin from dihydroflavonol to produce cyanidin. Anthocyanin synthesis depends on *F3′H* gene expression, as shown by tea plants (*Camellia sinensis*) and Senecio cruentus [36,37]. In our study, two *F3H* and one *F3′H* gene were found to have an enhanced expression in PCT than in GCX (Figure 3). As a result, we hypothesized that *F3H* and *F3′H* were involved in the regulation of anthocyanin synthesis in PCT. It has been shown that DFR can catalyze the production of leucodelphinidin, leucocyanidin, and leucopelargonidin, which are all antecedents to *ANS*. Previous studies showed that relatively high DFR and ANS activity may provide more anthocyanin synthesis, and suppression of DFR and ANS resulted in the lack of anthocyanin in *Caryophyllales* plants [38]. Our study found higher expression of *ANS* and *DFR* in PCT than in GCX (Figure 3), and this is consistent with higher anthocyanin levels in PCT. Anthocyanins are transported into the vacuole by a protein encoded by the GST gene [39,40,41,42]. The *GSTF12* (*BraA10g022830.3.5C*) expression was higher in PCT than in GCX (Figure 4), and according to our hypotheses, PCT expression of *GSTF12* allows more anthocyanins to be transported to the vacuole for immobilization due to the higher expression levels of *GSTF12*.

There are several transcription factors considered to be involved in the regulation of the anthocyanin biosynthesis and their transcriptional levels, which include bHLH, MYB, WD40, and members of a few other transcription factor families. It has been demonstrated that transcription factors play an important role in the regulation of genes involved in the anthocyanin biosynthetic pathway in vegetables, fruits, tea, and flowers [10,43,44,45]. A previous study reported that MYB/bHLH/WD-repeat transcriptional developments mainly regulate late biosynthesis genes (LBGs) in flavonoid biosynthesis [46,47]. In PAP1-overexpressing *Arabidopsis* leaves, flavonoid pigments are upregulated through the phenylpropanoid pathway, resulting in an increased expression of LBGs and early biosynthetic genes (EBGs) [48]. From the above research, it is suggested that transcription factors might not only regulate LBG genes but also other genes involved in the flavonoid pathway. Therefore, it is vital to analyze transcription factor expression levels to gain a deeper understanding of anthocyanin synthesis regulation. As a result of our study, we identified that *BrMYB114* has a significantly higher level of expression in PCT (Figure 3), suggesting that *BrMYB114* is involved in the regulation of anthocyanin biosynthesis. By overexpressing *BrMYB114* in *N. benthamiana*, higher anthocyanin content was observed in leaves of *N. benthamiana* overexpressing *BrMYB114* than control leaves transformed with the empty expression vector. Whether early biosynthesis genes or late biosynthesis genes are regulated by *BrMYB114* contributing to anthocyanin accumulation needs to be resolved in a further study.

## 4. Materials and Methods

### 4.1. Plant Materials

Flowering Chinese cabbage plants, including a purple inbred line (Zicaitai, PCT) and a green inbred line (Futiancaixin, GCX) were obtained from the Vegetable Research Institute, Guangdong Academy of Agricultural Sciences, Guangzhou, China. Plants were planted in pots (130 mm diameter × 140 mm deep) filled with soil (Shenglvyuan, Guangzhou, China) and grown inside the greenhouse at the Vegetable Research Institute, Guangdong Academy of Agricultural Sciences, at a 16-h light/8-h dark photoperiod at 25 °C, and the relative humidity was approximately 60%. Epidermal tissue was separated from the stems of plants with a blade, directly frozen in liquid nitrogen, and quickly stored at −80 °C until analysis. Both cultivars were planted in three biological replicates, each containing five plants.

### 4.2. Quantification of Anthocyanin Content

Anthocyanin contents were analyzed as described previously [49]. Briefly, the samples were frozen in liquid nitrogen, chopped, and then ground to a fine powder. The ground tissue was homogenized in methanol containing 1% HCl and incubated at 4 °C for 24 h. Anthocyanin contents were quantified by a spectrophotometer measuring the absorbance at 530 nm and 657 nm.

### 4.3. RNA Extraction, Library Construction, RNA Sequencing, and Assembly

For RNA isolation, the total samples were chopped and ground to a powder in liquid nitrogen, and 100 mg of plant material was used for extraction of RNA. Total RNA was isolated from the samples by using an RNAprep Pure Plant Kit (Tiangen, Beijing, China). The quality and integrity of the extracted RNA were determined using an Agilent 2100 bioanalyzer (Agilent, Santa Clara, CA, USA) and RNase-free agarose gel electrophoresis, respectively. An Illumina sequencing library was constructed using a Hieff NGS, Ultima Dual-mode mRNA Library, and Prep Kit (Yeasen Biotechnology (Shanghai) Co., Ltd., Shanghai, China). After the RNA samples passed the quality test, eukaryotic mRNA was enriched using oligo-dT magnetic beads. Subsequently, a fragmentation buffer was added to randomly fragment the mRNA. The fragmented mRNA was used to synthesize cDNA, and the newly synthesized cDNA was purified. The purified double-stranded cDNA was end-repaired, and an A tail was attached to the cDNA via a sequencing connector. AMPure XP beads (Yeasen Biotechnology (Shanghai) Co., Ltd.) were used for the size selection of the fragments, and PCR enrichment was used to generate the cDNA library. Six libraries from two groups were sequenced and analyzed on the Illumina NovaSeq6000 platform. After removing the adapter sequences and the low-quality reads, the clean reads were mapped to the reference genome (Brassicaceae Database).

### 4.4. RNA Sequencing Data Analysis and Annotation

To identify the functions of the assembled genes, a BLAST homology search was conducted on the database of non-redundant protein sequences in the (NCBI) database, as well as the Swiss-Prot, Clusters of Orthologous Groups of Proteins (COG), Gene Ontology (GO), Protein Family (Pfam), and Kyoto Encyclopedia of Genes and Genomes (KEGG) databases. Fragments per kilobase of transcript per million mapped reads (FPKM) were used for measuring the expression levels of genes. |log2 FC| ≥ 1 and FDR < 0.01 were used as criteria to screen (DEGs). The DEGs were further subjected to KEGG and GO enrichment data validations.

### 4.5. qRT-PCR Gene Expression Analysis

qRT-PCR was performed by using a Biomarker Plant Total RNA Extraction Kit (Biomarker, Beijing, China) for the extraction of RNA and a HiScript III 1st Strand cDNA, Synthesis Kit with the gDNA wiper (Vazyme Biotech, Nanjing, China) for the synthesis of cDNA. qRT-PCR primers were designed using Primer Premier Software 5.0 (Table S7). The qRT-PCR was performed in a Bio-Rad CFX96, PCR machine (Bio-Rad Laboratories, Hercules, CA, USA) using a Vazyme Master Mix SYBR qPCR (Vazyme Biotech, China). PCR amplification was carried out in a total volume of 10 μL (5 μL 2xChamQ Universal SYBR qPCR Master Mix, 3.4 μL ddH_2_O, 0.3 μL downstream primer, 0.3 μL upstream primer, and 1 μL cDNA template). The levels of relative gene expression were quantified by using the 2^−ΔΔCT^ method with the GAPDH gene as the reference control.

### 4.6. Transient Overexpression in Nicotiana benthamiana Leaves

Agrobacterium colonies harboring the target construct were selected and inoculated into a 20 mL LB culture medium containing the corresponding antibiotics. As a result of centrifugation, the cells were further resuspended in buffer (10 mM MES, 10 mM MgCl_2_, 150 mM acetosyringone, pH 5.6), and then by adding infiltration buffer (10 mM MES, 10 mM MgCl_2_, 150 mM acetosyringone, pH 5.6), OD600 was adjusted to 1.0. The Agrobacterium cultures were injected using a needleless syringe into the leaf epidermis of 4- to 6-week-old *N. benthamiana* plants, and Agrobacterium transformed with pGreen II 0029 62-SK vector (SK) as controls. *N. benthamiana* plants were grown in a growth chamber along with a relative photoperiod of 16 h of light and 8 h of darkness. After 7 days, the phenotypes of the leaves were photographed, and the anthocyanin contents were measured.

### 4.7. Statistical Analysis

Three biological repetitions were set up for each experiment, and all data were expressed as mean ± SD. GraphPad Prism8.0.lnk was used for *t*-tests. Groups with significant differences were set at *p* < 0.05.

## 5. Conclusions

In the present study, we provide a deeper insight into the anthocyanin biosynthetic pathway from the comparative transcriptome analysis of flowering Chinese cabbages. A total of 25 DEGs were identified that are related to flavonoid and anthocyanin biosynthesis pathways, indicating that they play an important role in anthocyanin biosynthesis in flowering Chinese cabbage. Further, we explained the function of *BrMYB114* in anthocyanin synthesis and showed that *BrMYB114* is an important component of anthocyanin synthesis.

## Data Availability

Data presented in this study are available upon request from the corresponding author.

## Supplementary Materials

The following supporting information can be downloaded at: https://www.mdpi.com/article/10.3390/ijms241813951/s1.
