# Supplementary material for: Comparative Transcriptome Analysis of Purple and Green Flowering Chinese Cabbage and Functional Analyses of BrMYB114 Gene
Source: Int J Mol Sci. 2023 Sep 11;24(18):13951. doi: 10.3390/ijms241813951 (PMC10531404; doi:10.3390/ijms241813951)
Supplement: Supplementary file 1 [file ijms-24-13951-s001.zip › Supplementary Materia figures.pdf]

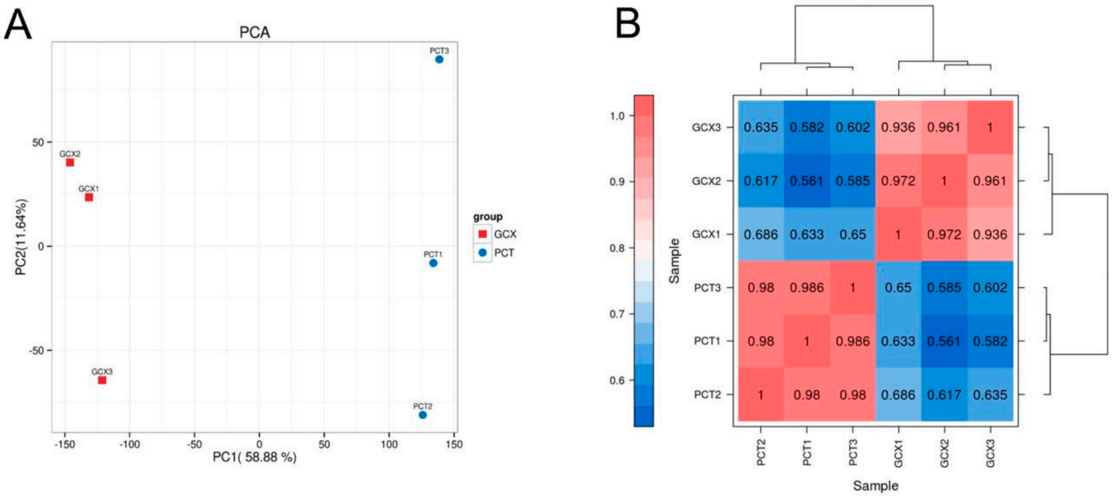

Figure S1 PCA analysis and Intergroup correlation analysis of PCT and GCX. Principal component analysis of each group samples (A). Intergroup correlation analysis of each group samples (B).

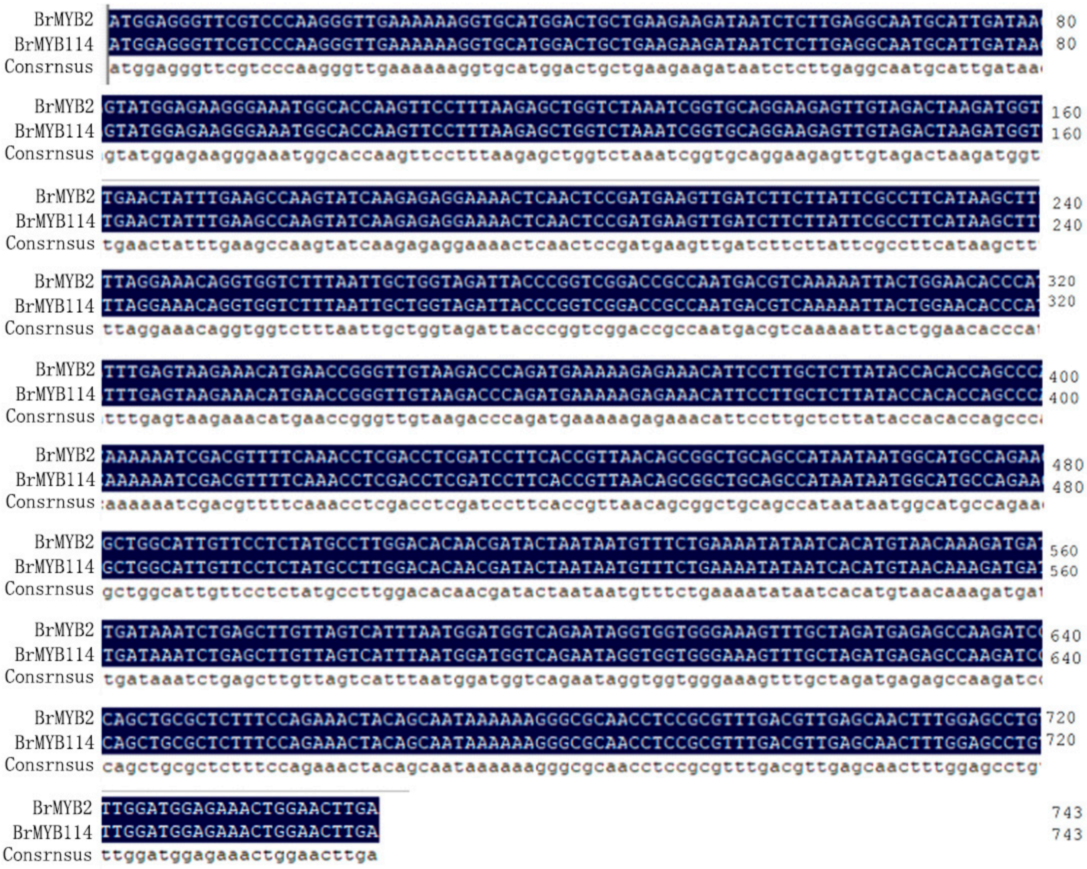

Figure S2 DNA sequence alignment of BrMYB2 and BrMYB114
